# *MTTP-*297H polymorphism reduced serum cholesterol but increased risk of non-alcoholic fatty liver disease-a cross-sectional study

**DOI:** 10.1186/s12881-015-0242-6

**Published:** 2015-10-12

**Authors:** Pi-Jung Hsiao, Mei-Yueh Lee, Yeng-Tseng Wang, He-Jiun Jiang, Pi-Chen Lin, Yi-Hsin Connie Yang, Kung-Kai Kuo

**Affiliations:** Division of Endocrinology and Metabolism, Department of Internal Medicine; Kaohsiung Municipal Siaogang Hospital, Kaohsiung Municipal Ta-Tung Hospital, Kaohsiung Medical University Hospital, Kaohsiung, Taiwan; School of Medicine, College of Medicine, Kaohsiung Medical University, 100 Tzyou 1st Rd, Kaohsiung, 807 Taiwan; Department of Biochemistry, College of Medicine, Kaohsiung Medical University, Kaohsiung, Taiwan; School of Pharmacy, College of Pharmacy, Kaohsiung Medical University, Kaohsiung, Taiwan; Division of Hepatobiliopancreatic Surgery, Department of Surgery, Kaohsiung Medical University Hospital, Kaohsiung, Taiwan

**Keywords:** Microsomal triglyceride transfer protein (MTP), Apob-containing lipoproteins, LDL-C, Non-HDL-C, Non-alcoholic fatty liver disease (NAFLD), Q297H polymorphism

## Abstract

**Background:**

Microsomal triglyceride transfer protein (MTP) works to lipidate and assemble the apoB-containing lipoproteins in liver. It closely links up the hepatic secretion of lipid to regulate serum lipid and atherosclerosis. Cases of *MTTP* gene mutation is characterized by abetalipoproteinemia and remarkable hepatic steatosis or cirrhosis. Several *MTTP* polymorphisms have been reported relating to metabolic syndrome, hyperlipidemia and steatohepatitis. We supposed the regulation of serum lipids and risk of non-alcoholic fatty liver disease (NAFLD) formation may be modified by individual susceptibility related to the *MTTP* polymorphisms.

**Methods and results:**

A cross-sectional population of 1193 subjects, 1087 males and 106 females mean aged 45.9 ± 8.9 years, were enrolled without recognized secondary hyperlipidemia. Fasting serum lipid, insulin, and non-esterified fatty acid were assessed and transformed to insulin resistance index, HOMA-IR and Adipo-IR. After ruling out alcohol abuser, non-alcoholic fatty liver disease (NAFLD) was diagnosed by abdominal ultrasound. Five common *MTTP* polymorphisms (promoter -493G/T, E98D, I128T, N166S, and Q297H) were conducted by TaqMan assay. Multivariate regression analysis was used to estimate their impact on serum lipid and NAFLD risk. Assessment revealed a differential impact on LDL-C and non-HDL-C, which were sequentially determined by the Q297H polymorphism, insulin resistance, body mass index and age. Carriers of homozygous minor allele (297H) had significantly lower LDL-C and non-HDL-C but higher risk for NAFLD. Molecular modeling of the 297H variant demonstrated higher free energy, potentially referring to an unstable structure and functional sequence.

**Conclusion:**

These results evidenced the *MTTP* polymorphisms could modulate the lipid homeostasis to determine the serum lipids and risk of NAFLD. The *MTTP* 297H polymorphism interacted with age, insulin resistance and BMI to decrease serum apoB containing lipoproteins (LDL-C and non-HDL-C) but increase the risk of NAFLD formation.

**Electronic supplementary material:**

The online version of this article (doi:10.1186/s12881-015-0242-6) contains supplementary material, which is available to authorized users.

## Background

Microsomal triglyceride transfer protein (MTP) resides in the microsomes of hepatocytes and enterocytes as a chaperon to preferentially transfer neutral lipids (triglycerides and cholesterol ester). It is responsible for the assembly and secretion of triglyceride rich apoB-containing lipoproteins such as chylomicron, very low density lipoproteins (VLDL-C), and low density lipoproteins (LDL-C) [1, 2]. Abetalipoproteinemia (ABL) is a rare disease, characterized by absence or very low apoB-containing lipoproteins in plasma and remarkable hepatic steatosis or cirrhosis, which is attributable to decreased lipid secretion from the liver and caused by *MTTP* gene mutation [3, 4].

Non-alcoholic fatty liver disease (NAFLD) is strongly concomitant with obesity, type 2 diabetes mellitus and hypertriglyceridemia, and also regarded as a hepatic manifestation of metabolic syndrome [5]. An imbalance of fatty acid homeostasis may contribute to the development of NAFLD, including excess dietary fat intake, increased fatty acid influx, de novo lipogenesis, decreased β-oxidation of fatty acid or reduced export of triglyceride-rich lipoproteins. The functional variability of MTP is linked to the development of NAFLD, raised lipid and risk of atherosclerotic cardiovascular disease (CAD) [6, 7]. However, no large scale studies have been performed to provide solid evidence for the association among *MTTP* genetic polymorphisms, serum lipid level and NAFLD formation. Early recognition of genetic *MTTP* polymorphisms that confer a higher risk of developing NAFLD would improve the clinical management of this growing fast disease.

The MTP protein is a heterodimer composed of M-subunit (sized ~97 kDa) and P subunit (protein disulfide isomerase, PDI, sized ~55 kDa) in a 1:1 stoichiometry by non-covalent interactions. The MTP protein exhibits three major structural domains responsible for different functions. The N-terminal β-barrel (β^N^, residues 22-297) of human MTP binds with apoB, the central α-helical domain (residues 298-603) interacts with PDI and apoB, and the two C-terminal β-sheet domains (β^C^ and β^A^, residues 604-894) mediate lipid-binding and transfer activity [2, 8].

Almost all of the reported *MTTP* mutations residue in the α-helical and C-terminal functional domains, which mainly mediate the interaction of PDI and apoB to regulate the lipid-binding or lipid-transfer activity, respectively. Most of these mutations are deletions, premature stop codons and splice mutation and always cause virtual absence of apoB with health-threatening or even life-threatening sequelae, such as failure to thrive, fat malabsorption, neuropathy or myopathy in later life [9, 10]. However in literature review, *MTTP* polymorphisms on the promoter region and N-terminal of β-barrel (22-297) may modulate the MTP activity but not causing ABL [11–13]. The *MTTP* gene (4q24) is polymorphic with numerous variants and remains in linkage disequilibrium. Previous reports have demonstrated subjects with *MTTP* promoter -493G/T or I128T polymorphisms are susceptible to develop metabolic syndrome, hyperlipidemia, more oxidative stress, ischemic heart disease, β-cell dysfunction and non-alcoholic steatohepatitis [11–15]. But, some studies have controversial and inconsistent results.

The human *MTTP* gene is suppressed by insulin but enhanced by a high fat or cholesterol-enriched diet [2, 3, 16]. We assumed genetic effect of the *MTTP* polymorphisms may interact with the metabolic regulators, such as age, sex, body mass index (BMI), and insulin resistance, to regulate the lipid homeostasis and hepatic steatosis. This study, focusing on common polymorphisms over promoter (-493G/T) and N-terminal β-barrel (residues 22-297) of *MTTP*, was tried to estimate and compare the genetic impact on serum lipids and NAFLD, adjusted by the above metabolic regulators. The results could eventually be applied in clinic for identifying high-risk candidates for hyperlipidemia and NAFLD.

## Methods

### Selection criteria

This cross-sectional study was designed and conducted in adherence to the STROBE guidelines. The purposes, rationale, methodology, and risks to the participants including psychosocial stress were reviewed, supervised and approved by the Institutional Review Board of Kaohsiung Medical University Hospital (KMUH-IRB-980323). The subjects, who were recruited from health check-ups at the Department of Preventive Medicine at KMUH, had their serum stored in the tissue bank after informed consent obtained. Serum of all enrolled participants was provided from the tissue bank after de-identification of their names and personal characteristics. Participants with recognized secondary dyslipidemia, including known diabetes, nephrotic syndrome, Cushing’s syndrome, hypothyroidism, chronic liver disease, alcoholism or current users of lipid-lowering agents were evaluated in a detailed medical history review by an experienced physician and excluded from this study. Ultimately a cross-sectional population of 1193 subjects (1087 male and 106 female, mean age 45.9 ± 8.9 years) was enrolled randomly within half a year. The age distribution of the study population ranged from 16 to 88 years, with 95 % aged between 25 and 65 years.

### Biochemistry measurements and evaluation of fatty liver

Fasting blood samples were assessed by multichannel auto-analyzer for serum glucose, aspartate aminotransferase (AST), alanine aminotransferase (ALT), total cholesterol, triglyceride, HDL-cholesterol (HDL-C), and LDL-cholesterol (LDL-C). Fasting serum insulin and non-esterified fatty acid (NEFA) were measured by commercial RIA and ELISA kits as in our previous experiments. The objective and quantitative insulin resistance indexes were obtained and expressed as HOMA-IR (= insulin (μU/mL) × glucose (mmol/L)/22.5) and Adipo-IR index (= fasting insulin (μU/mL) × NEFA (μmol/L)).

Evaluation for fatty liver disease was performed by abdominal B-mode ultrasound (3.5 MHz convex transducer, Toshiba SSA-250, Tokyo, Japan). Diagnosis and grading of the fatty liver was carried out by well-trained hepatologists using the standard ultrasonography criteria in our hospital to achieve inter-individual consistency.

### Search strategy

Blood samples were collected after overnight fasting for DNA extraction and routine biochemistry. TaqMan technology was used to detect the sequence variants of five common microsomal triglyceride transfer protein (*MTTP*) polymorphisms (*rs1800591* for promoter -493G/T; *rs2306986* for Glu98Asp (E98D), *rs3816873* for Ile128Thr (I128T), *rs3792683* for Asn166Ser (N166S), *rs2306985* for Gln297His (Q297H)). To achieve significance from our limited sample size, non-synonymous polymorphisms with minor allele frequency more than 10 % in the Han population were chosen according to the SNP reference in NCBI GenBank website. The sequence variants were further analyzed by the ABI PRISM 7500^R^ (Applied Biosystems, Roche, Taipei, Taiwan) detection system.

### Molecular modeling assay

The I-TASSER server was used for high-resolution modeling of structural and functional predictions for the MTP protein by submitting the amino acid sequences (NCBI accession number AAI 25112.1) to obtain the initial 3D model [17]. The solvated protein structure inserted in a Tip3p water box was applied for the molecular dynamics (MD) simulations and performed in the canonical ensemble with a simulation temperature of 310 K by Verlet integrator with an integration time step of 0.002 ps and SHAKE constraints of all covalent bonds involving hydrogen atoms. In the electrostatic interactions, atom-based truncation was performed using the PME method, and the switch van der Waals function was used with a 2.00 nm cutoff for atom-pair lists. The structure was minimized for 100,000 conjugate gradient steps, and then subjected to a 100 ns isothermal, constant volume MD simulation by the Amber 14 (pmemd.cuda) program. The final model was derived using the SDM server to predict the effects of mutations (H297Q) on protein stability and visualized with PyMOL [18].

### Statistical analysis

All of the statistical analysis was conducted using the SPSS 19.0 statistical package for Windows (SPSS Inc., Chicago, IL, USA). Allele frequencies were estimated by direct counting, while each genotypic distribution was assessed for the Hardy-Weinberg equilibrium by chi-square test. Haploview 4.2^TM^ software (Broad Institute, Cambridge, MA, USA) was used to reconstruct the haplotype blocks. Continuous variables were expressed as mean ± standard deviation (SD). The normal distributed variables were compared between groups using an independent *t* test. The Nonparametric Man-Whitney rank-sum test was used to analyze non-normally distributed variable of serum triglyceride. Multiple linear regression analysis was employed using serum LDL-C, non-HDL-C, and triglyceride as a dependent variable, while multiple logistic regression analysis was employed using the presence of NAFLD as a dependent variable to recruit BMI, HOMA-IR, Adipo-IR and the genotypes of *MTTP* polymorphisms as independent variables based on significance in univariate analyses and regression models. The hazard of dyslipidemia and of NAFLD by the haplotype effects were further analyzed by the SAS 9.3 system (SAS Institute Inc., Cary, NC, USA). There were 19 genotype combinations for these above polymorphisms. To avoid bias, we excluded 9 combinations because of small subject numbers (≤4) and missing data. Principally, genotype of the GG: GT: TT was simplified as number 1: 2: 3. All the genotype combination (haplotype) was according to the sequence of G-493T/E98D/I128T/N166S/Q297H polymorphisms. Frequency of the most popular genotypes combination, -493GG/E98/I128/S166/Q297H simply indicated as 11132 (GG/GG/TT/GG/GC), was 22.43 % as reference control to compare the relative risk of other combinations for metabolic abnormalities.

## Results

**Fig. 1:**
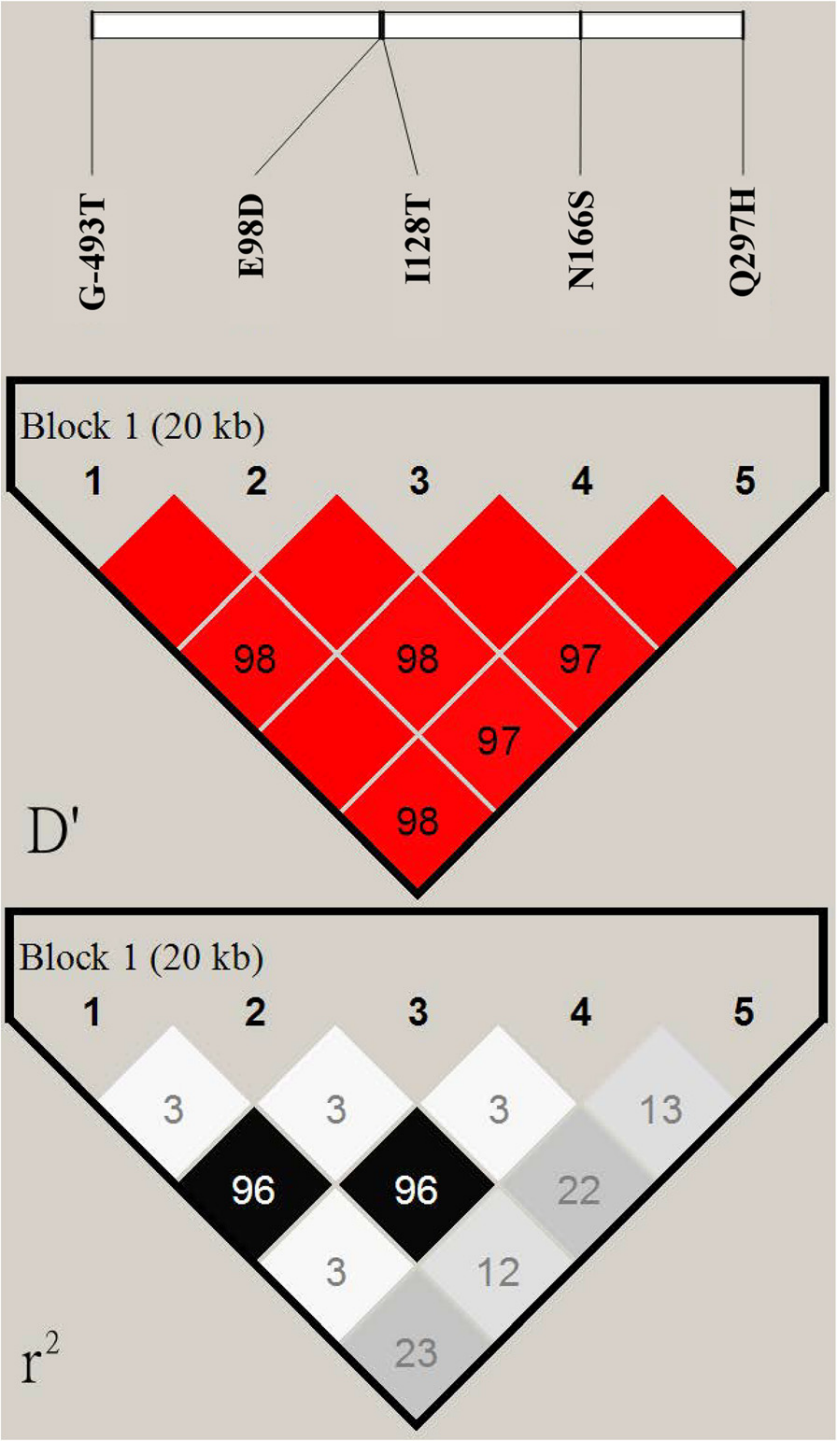
Haplotype block map for five common *MTTP* polymorphisms. *Top* plot: The linkage disequilibrium between two SNPs is standardized as D’. The *dark grey* cells indicate strong linkage disequilibrium among these SNPs. *Bottom* plot: The gray-scale spectrum (from left to right) indicates pairwise *r* ^2^ (%) values ranging from *black* (*r* ^2^ = 1) to *white* (*r* ^2^ = 0)

Minor allele frequencies of the five common non-synonymous *MTTP* polymorphisms were: promoter -493 (G: T = 84: 16 %), E98D (G: C = 84.1: 15.9 %), I128T (T: C = 86.3: 15.9 %), N166S (A: G = 84: 16 %) and Q297H (G: C = 59.7: 40.3 %). All of the genotype distributions were tested in Hardy-Weinberg equilibrium (Table 1). There is strong linkage disequilibrium with allelic association in all pairwise combination (pairwise D’ greater than 0.97) among these five polymorphisms (Fig. 1). Cellular MTP is an essential chaperone to transfer triglycerides and cholesterol esters for the biosynthesis of apo-B-containing lipoprotein particles. Therefore, the serum lipid level determined by *MTTP* polymorphisms was classified by cholesterol and triglyceride for further analysis. As shown in Table 2, subjects with CC genotype (297H) had significantly lower serum LDL-C than those with GG+GC genotypes.

**Table 1:** Distribution of *MTTP* genotypes

NCBI references	Gene polymorphisms	Allele change	Genotypes	Distribution (numbers, %)
rs1800591	(promoter) G-493T	G/T	GG: GT: TT	882: 273:29 (74.5 %: 23.1 %: 2.4 %)
rs2306986	E98D	G/C	GG: GC: CC	809: 318: 25 (70.2 %: 27.6 %: 2.2 %)
rs3816873	I128T	T/C	TT: TC: CC	878: 270: 26 (74.8 %: 23.0 %: 2.2 %)
rs3792683	N166S	A/G	AA: AG: GG	818: 327: 24 (70.0 %: 28.0 %: 2.0 %)
rs2306985	Q297H	G/C	GG: GC:CC	414: 566: 193 (35.3 %: 48.3 %: 16.4 %)

Genotype distribution was in Hardy-Weinberg equilibrium tested by chi-square test

**Table 2:** Comparison of the serum cholesterol, LDL-C and non-HDL-C with *MTTP* genotypes

Genotype	Cholesterol (mg/dl)	*P*	LDL (mg/dl)	*P*	Non-HDL (mg/dl)	*P*
(promoter) G-493T						
GG+GT (*n* = 1159)	180.7 ± 32.4	0.576	123.1 ± 33.1	0.391	129.3 ± 32.5	0.694
TT (*n* = 29)	184.1 ± 28.1		129.8 ± 29.7		132.3 ± 23.5	
Glu98Asp (E98D)						
GG+GC (*n* = 1132)	180.5 ± 31.9	0.551	123.2 ± 33.0	0.566	129.2 ± 32.3	0.719
CC (*n* = 25)	184.3 ± 28.5		127.7 ± 33.3		132.0 ± 2.7	
Iler128Thr (I128T)						
TT+TC (*n* = 1153)	180.6 ± 32.4	0.783	123.0 ± 33.1	0.384	129.3 ± 32.6	0.848
CC (*n* = 26)	182.4 ± 27.3		130.3 ± 31.6		130.9 ± 24.6	
Asn166Ser (N166S)						
AA+AG (*n* = 1150)	180.5 ± 32.2	0.489	122.9 ± 32.9	0.362	129.1 ± 32.4	0.543
GG (*n* = 25)	185.1 ± 29.2		130.1 ± 33.9		133.8 ± 27.9	
Gln297His (Q297H)						
GG+GC (*n* = 984)	180.9 ± 32.6	0.433	124.1 ± 33.7	0.047*	129.9 ± 33.1	0.282
CC (*n* = 193)	179.0 ± 29.6		118.5 ± 28.8		126.6 ± 28.4	

Data are shown as mean ± SD. Independent *t* test was used for statistical analysis. (* P < 0.05 indicates significant)

The fasting serum triglyceride, mainly carried in VLDL-C, is a major component of metabolic syndrome and highly contributes to insulin resistance. We compared these metabolic abnormalities with *MTTP* polymorphisms by univariate analysis (Table 3). Subjects with CC genotype (98D) and GG genotype (166S) apparently had significantly lower BMI. Subjects with TT genotype of promoter G-493T, CC genotype (98D), CC genotype (128T) and GG genotype (166S) had significantly lower serum triglyceride. The lower Adipo-IR was correlated well with promoter G-493T and 128T. However, the Q297H genotype was not correlated with these metabolic parameters.

**Table 3:** Comparison of the metabolic parameters with *MTTP* genotypes

Genotype	BMI (kg/m^2^)	*P*	TG (mg/dl)	*P*	HOMA-IR	*P*	Adipo-IR	*P*
(promoter) G-493T								
GG+GT (*n* = 1155)	24.5 ± 3.3	0.527	138.3 ± 123.9	0.039*	1.09 ± 2.29	0.572	2.46 ± 3.84	0.014*
TT (*n* = 29)	24.7 ± 2.5		117.1 ± 49.7		0.83 ± 0.72		1.61 ± 1.32	
Glu98Asp (E98D)								
GG+GC (*n* = 1127)	24.5 ± 3.3	0.050*	137.0 ± 120.9	0.001*	1.09 ± 2.31	0.044*	2.48 ± 3.87	0.436
CC (*n* = 25)	22.6 ± 2.3		101.3 ± 43.2		0.61 ± 1.02		1.78 ± 2.98	
Ile128Thr (I128T)								
TT+TC (*n* = 1148)	24.5 ± 3.3	0.640	138.1 ± 123.6	0.040*	1.08 ± 2.29	0.140	2.46 ± 3.86	0.017*
CC (*n* = 26)	24.8 ± 2.6		114.9 ± 51.9		0.833 ± 0.75		1.57 ± 1.38	
Asn166Ser (N166S)								
AA+AG (*n* = 1145)	24.5 ± 3.3	0.006*	138.6 ± 123.9	0.001*	1.05 ± 1.92	0.301	2.43 ± 2.75	0.442
GG (*n* = 24)	22.7 ± 2.3		102.8 ± 44.3		0.62 ± 1.04		1.77 ± 2.98	
Gln297His (Q297H)								
GG+GC (*n* = 984)	24.5 ± 3.4	0.913	136.9 ± 124.1	0.656	1.09 ± 2.43	0.451	0.57 ± 0.23	0.337
CC (*n* = 193)	24.5 ± 2.9		141.2 ± 116.2		0.92 ± 0.98		0.58 ± 0.25	

Data are shown as mean ± SD

Independent *t* test was used for statistical analysis of body mass index (BMI) and insulin resistance (HOMA-IR and Adipo-IR)

Mann–Whitney *U* test was used to analyze triglyceride (TG). (* P < 0.05 indicates significant)

The relative impact of the *MTTP* genotypes and their interaction with confounding risks on serum LDL-C were further tested by multivariate regression analysis (Table 4). The CC genotype (297H) had a significantly greater impact on reduced serum LDL-C than age and BMI. Moreover, serum non-HDL-C was significantly negatively determined by homozygous CC genotype (297H) and HOMA-IR, while positively regulated by Adipo-IR, BMI and age in that sequence (Table 5). By multivariate analysis, the significant association of *MTTP* polymorphisms with serum triglyceride was overpowered by the impact of insulin resistance (HOMA-IR and Adipo-IR) and BMI (Additional file 1). These results suggest insulin resistance and BMI significantly contribute more to serum triglyceride than the genetic effect of *MTTP* polymorphisms.

**Table 4:** Risk impact and interaction of the *MTTP* genotypes on serum LDL-C

Independent variables	Parameter estimates (B)	95 % Confidence interval		*P*
		Lower vs Upper bound		
Age	0.430	0.150	0.710	0.003*
Sex (male vs female)	−7.279	−43.699	29.141	0.695
BMI	1.448	0.636	2.261	<0.0001*
HOMA-IR	−1.189	−3.543	1.166	0.322
Adipo-IR	1.414	−0.168	2.996	0.080
E98D (CC vs GG+GC)	−32.004	−96.849	32.840	0.333
I128T (CC vs TT+TC)	12.878	−7.070	32.827	0.205
N166S (GG vs AA+AG)	35.276	−27.455	98.007	0.270
Q297H (CC vs GG+GC)	−10.802	−17.822	−3.782	0.003*

Multiple linear regression analysis was applied using serum LDL-C as dependent variable adjusted by age, sex, BMI, HOMA-IR, Adipo-IR and *MTTP* genotypes based on significance in univariate analysis and regression models. (*P < 0.05 indicates significant)

**Table 5:** Risk impact and interaction of the *MTTP* genotypes on serum non-HDL-C

Independent variables	Parameter estimates (B)	95 % Confidence interval		*P*
		Lower vs Upper bound		
Age	0.510	0.247	0.772	<0.0001*
Sex (male vs female)	−9.122	−43.247	25.035	0.6006
BMI	1.343	0.581	2.105	0.0011*
HOMA-IR	−4.397	−6.606	−2.189	<0.0001*
Adipo-IR	4.157	2.673	5.641	<0.0001*
E98D (CC vs GG+GC)	−23.150	−83.966	37.666	0.455
I128T (CC vs TT+TC)	6.400	−12.309	25.110	0.502
N166S (GG vs AA+AG)	23.984	−34.850	82.818	0.424
Q297H (CC vs GG+GC)	−6.802	−13.386	−0.218	0.043*

Multiple linear regression analysis was applied using serum non-HDL-C as dependent variable adjusted by age, sex, BMI, HOMA-IR, Adipo-IR and *MTTP* genotypes based on significance in univariate analysis and regression models. (*P < 0.05 indicates significant)

The frequency of NAFLD in our adult population was 58.5 % (*n* = 658), which included 6.4 % with severe NAFLD. As shown in Table 6, the risk of developing NAFLD was significantly correlated with the CC genotype (297H), BMI and Adipo-IR in that order. The risk impact on NAFLD was higher by the genetic effect of 297H polymorphism than BMI or insulin resistance. Our results revealed that carriers of homozygous 297H had significantly lower serum LDL-C and non-HDL-C but a greater risk of developing NAFLD adjusted by insulin resistance, BMI and age. To investigate the cumulative effect of these polymorphisms, the lipid and metabolic parameters were compared among the 10 genotype combinations (Additional file 2). This revealed that females have significantly lower LDL-C, non-HDL-C, BMI, aspartate and alanine transaminase (AST and ALT) but higher HDL-C than males. This may be implicated in a protective effect for atherosclerosis progression in females. The haplotype 21233 (GT/GG/TC/GG/CC) carriers had significantly lower serum cholesterol, LDL-C and non-HDL-C than controls with a greater impact from genetic effect than age and gender. Carriers of the haplotype 11133 (GG/GG/TT/GG/**CC**), especially having the C allele of Q297H, significantly displayed increment of AST (2.2 IU/L) and ALT (3.8 IU/L) and hazard for fatty liver formation (odds ratio 1.68) compared with control.

**Table 6:** Risk impact and interaction of the *MTTP* genotypes on non-alcoholic fatty liver disease (NAFLD)

Independent variables	Odds ratio (95 % confidence interval)	*P*
Age	0.991 (0.973–1.009)	0.313
Sex	1.252 (0.071–21.976)	0.878
BMI (kg/m^2^)	1.370 (1.288–1.457)	<0.0001*
HOMA-IR	0.895 (0.694–1.154)	0.391
Adipo-IR index	1.266 (1.175–1.365)	<0.0001*
Promoter (TT vs GG+GT)	0.588 (0.194–1.785)	0.349
E98D (CC vs GG+GC)	0.711 (0.040–12.723)	0.816
N166S (GG vs AA+AG)	1.028 (0.057–18.483)	0.985
Q297H (CC vs GG+GC)	1.682 (1.102–2.568)	0.016*

Multiple logistic regression analysis was applied using NAFLD as a dependent variable adjusted by age, sex, BMI, HOMA-IR, Adipo-IR and *MTTP* genotypes based on significance in univariate analysis and regression models. (*P < 0.05 indicates significant)

**Fig. 2:**
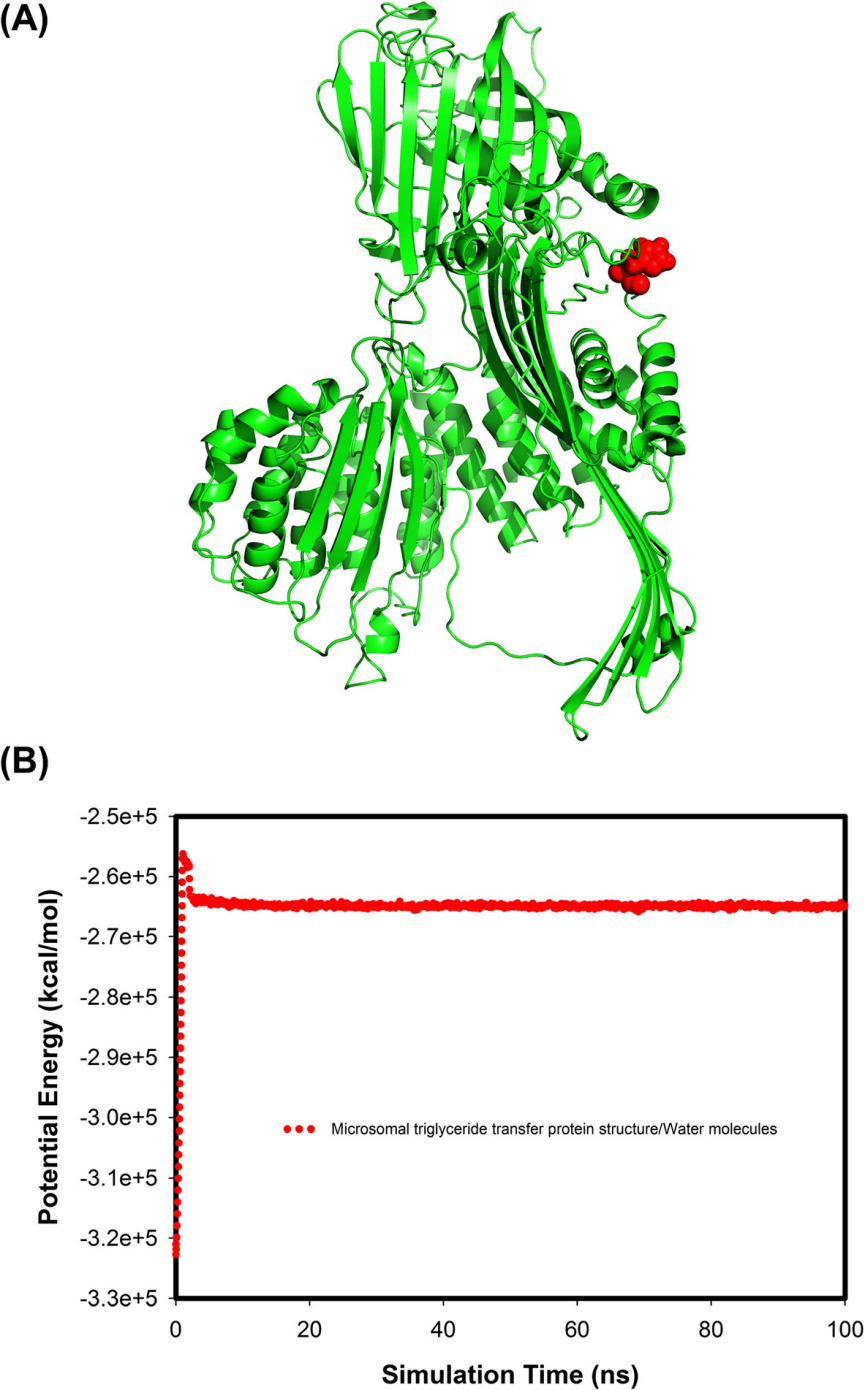
Predicted molecular modelling structure and potential energy plots of the microsomal triglyceride transfer protein (MTP). **a** 297H amino acid resides at the end of β-barrel shown in a ball/stick model. **b** The potential energy for MTP in Tip3 water molecules demonstrated higher free energy and a less stable structure for 297H vs Q297

Predicted molecular modeling of the MTP structure and potential energy equilibrium were shown in Fig. 2. The potential energy reached equilibrium after 2.80 ns over 100 ns md simulations. Calculation of the residue effect revealed a higher delta free energy 0.91 kcal/M (ΔG, Q297H) for 297H, indicating that 297H was less stable in protein structure than Q297 and that this may involve altered interaction of the β-barrel of MTP with apoB. Therefore, as a functional polymorphism of *MTTP*, the CC genotype (297H) may result in functional alteration of the MTP protein to decrease assembly and secretion of apoB-containing lipoproteins.

## Discussion

In this study, we observed that carriers of homozygous CC genotype (297H) had significantly lower serum LDL-C and non-HDL-C but greater risk of NAFLD. The genetic risk of 297H polymorphism for NAFLD was higher than BMI and insulin resistance. Our findings suggested that the *MTTP* Q297H polymorphism, substitution of glutamine by histidine in the N-terminal β-barrel, may have conformational change leading to a functional consequence in binding activity with apo B. Since the C allele of Q297H was quite prevalent (40.3 %) in our population, it may play an important role in NAFLD formation.

Promoter -493G/T polymorphism, conveyed by reporter gene assay in HepG2 cells, revealed marked enhancement (2-fold) of transcriptional activity by the T allele [19]. Previous studies reported that individuals carrying -493 TT had significantly lower LDL-C, triglyceride and triglyceride/apoB ratios in VLDL-C particles than carriers of the GG or GT [20]. Vohl et al. reported that genetic effect of the -493G/T polymorphism on lipid profile was modulated by gender, visceral adiposity and insulin level [21]. Our result was consistent with this finding in those -493 TT carriers had significantly lower serum triglyceride and insulin resistance than GG or GT carriers. In KORA study cohort, carriers with homozygous minor allele (297Q) displayed a decrement for BMI and total cholesterol in females but not in males [22]. The homozygous 297Q and heterozygous -493GT carriers, reported by Talmud et al., were found to have higher serum triglyceride and raising effect on apoB levels [23]. Our results displayed carriers of homozygous minor allele (297H) to have lower apoB-containing lipoproteins, LDL and non-HDL level, but higher risk for NAFLD. However, some studies have reported to the contrary [24]. A meta-analysis of 11 clinical case–control studies also found that carriers of G versus T allele might increase individual susceptibility to NAFLD in both the Caucasian and non-Caucasian populations [25]. Our study recruited a relatively comparable sample size but had observed different results. Based on our result, metabolic regulators such as insulin resistance or body mass index did modulate the phenotypic manifestation of *MTTP* polymorphisms. As there was strong linkage disequilibrium between these common polymorphisms, we thought these inconsistent results may be interrelated to the potential heterogeneity of control, interference of the haplotypes and neglecting these confounders. So, we proposed all the possible confounders are obligatory to recruit in multivariate analysis to verify the real genetic effect.

Formation of the VLDL-C involves two-steps process. First, primordial apoB precursors are formed to fuse with small luminal lipid droplets, which are mainly mediated by the MTP protein. Second, the bulk neutral lipids, composed of triglyceride, cholesterol ester and phospholipids, are added to the preformed lipid droplets to form mature VLDL-C [26, 27]. The N-terminal domain of MTP (residues 22-297) interacts closely with apoB (residues 1-264) to determine the conformational and electrostatic properties of apoB and construct a suitable amphipathic lipid interface for further loading of the lipid core [8]. Residue Thr128 of MTP, located in a protruding loop at top of the β-barrel with a hydrophobic nature and more surface exposure, was less thermally stable and more easily cleaved in assembly with the apoB-MTP complex than the Ile128 residue. The I128T polymorphism, changing from an uncharged to a polar amino acid, was speculated to confer a change in electrostatic stability and alter the interactive binding activity of both proteins [28]. Our results have demonstrated strong linkage disequilibrium among these polymorphisms (-493G/T, E98D, I128T and N166S). Carriers with these minor alleles had significantly lower serum triglyceride in univariate analysis. This result implies that these missense *MTTP* polymorphisms may independently alter the binding or folding of MTP with apoB, and interfere the fusing with luminal lipid droplets. As the formed VLDL-C conveying less triglyceride (neutral lipid), it would rationally lead to lower serum NEFA, less visceral obesity and insulin resistance. However, our results revealed genetic impact of the *MTTP* polymorphisms on serum triglyceride was overpowered by insulin resistance and BMI. This result implicated that serum triglyceride was determined more by the insulin resistance and adiposity than the genetic effect of *MTTP* polymorphisms.

The “lipid-pocket” model is widely quoted to test the interplay of MTP with apoB, and MTP functions as a shuttle to fill the “lipid pocket” of the proposed LV-like apoB intermediate. There is one electrostatic binding site in region of N-terminal region (residues 22-297) of MTP and N-terminal region (residue 1-264) of apoB. The dense cluster of amphipathic β strands at the N-terminal end of MTP seems likely to be part of the binding site of MTP protein to shuttle monomeric triacylglycerol to apoB. So, the 297 residue resides in a critical location linking the β-barrel and α-helix of MTP [27, 29, 30]. This study revealed variant of the 297H versus Q297 demonstrated higher free energy equilibrium with an unstable protein structure. Hence, it may affect MTP and apoB binding, leading to malfunction of protein folding and lipidation with apoB.

The MTP protein is also expressed in cardiomyocytes and macrophages to determine lipid excretion from cells [3, 15]. Cases of ABL have been found to have severe cardiac lesions with excessive deposition of lipochrome pigment and extremely low apoB-containing lipoproproteins in serum. Tissue-specific *mttp* knockout mice have revealed cardiac lipid accumulation [31]. Cases of ABL and hypobetalipoproteinemia, even those exhibiting subtle differences in lipid phenotype, all demonstrate variable degrees of steatosis, elevated aminotransferase, hepatomegaly, steatohepatitis and profound fibrosis [4, 9, 32]. Taken together, functional MTP is important in the lipid metabolism to protect the heart and liver against lipotoxic injury. Moreover, pyrosequencing evidence has demonstrated that minor allele of the *MTTP* promoter (-493T/-164C) had lower transcriptional activity compared with the major alleles but expressed with some tissue discrepancy in heart, liver and macrophages [12, 33]. Our study demonstrated a novel finding, that carriers of the *MTTP* 297H had significantly lower LDL-C and non-HDL-C but higher risk for NAFLD. In theory, we speculated *MTTP* 297 polymorphism may alter binding activity of the MTP with apoB, leading to less secretion of apoB-containing lipoproteins. It may result in lower serum LDL-C, non-HDL-C and more lipid accumulation in liver. Even though carriers of 297H displayed lower atherogenic lipoproteins, more evidence is still needed to elucidate whether the risk for cardiac lipotoxicity or atherosclerosis is raised or reduced for the same given genotype.

As MTP is involved in the critical step regulating circulating lipid, MTP inhibition has emerged as a potential target and has been established to have efficacy in treating hypercholesterolemia patients [34]. The new therapies targeting apoB metabolism for high-risk patients with familial hypercholesterolemia, such as mipomersen to inhibit expression of hepatic apoB and lomitapide to block hepatic MTP activity, have demonstrated excellent efficacy for reducing atherogenic lipoproteins but have also significantly raised the risk of hepatic transaminase (10 %) and hepatic steatosis (18 %) in users. The changes in hepatic fat have been highly variable from patient to patient, ranging from a 1 % baseline to an average of 8.6 % at 6 months even up to 30 % in a few cases using MTP inhibitors [35–37]. Eventually, hepatic steatosis possibly predisposes to insulin resistance, metabolic syndrome, steatohepatitis and liver fibrosis [5]. Despite the US Food and Drug Administration (FDA) has approved both mipomersen and lomitapide, it still intensely mandates ensuring a maximal benefit-to-risk ratio of these drugs for clinically appropriate use [38]. Based on our results, the MTP inhibitors theoretically should not be used for subjects with familial hypercholesterolemia carrying *MTTP* 297H.

This study simultaneously compared the impact on serum lipid level and NAFLD formation by the interplay of *MTTP* polymorphisms and confounders. It is limited by the male dominant population even though our people have demonstrated highly prevalent NAFLD for male [39]. Abdominal ultrasound is generally applied in relatively large-scaled surveys as a noninvasive and convenient tool to diagnose NAFLD with acceptable sensitivity and specificity [5]. However, absent of severity grading of the hepatic steatosis in histology would weaken the clinical relevance of these genetic effects. Because both MTP and apoB are large proteins to interact in complex, clinical validation of the binding or transfer activity with apoB by these *MTTP* polymorphisms related changes is quite challenging. And our molecular modelling results may help elucidate the possible conformational changes and protein stability.

## Conclusions

In summary, our study demonstrated that carriers of the *MTTP* 297H significantly had lower apoB-containing lipoproteins (LDL-C, non-HDL-C) and greater risk of NAFLD adjusted by age, sex and insulin resistance. Genotype *MTTP* 297H may be an important and independent risk for NAFLD formation (odds ratio 1.68, 1.1–2.6) followed by BMI and Adipo-IR. Our study is the original report using a predicted molecular modeling to verify the potential functional alteration for this polymorphism. This result further evidenced the genetic risk in developing NAFLD.

## Additional files

Additional file 1:
**Risk impact and interaction of the MTTP genotypes on serum triglyceride.** (DOCX 21 kb) Additional file 2:
**Comparison of the biochemistry among the 10 haplotypes.** (DOCX 22 kb)

## Abbreviations

MTP: Microsomal triglyceride transfer protein
VLDL-C: Very-low density lipoprotein cholesterol
LDL-C: Low–density lipoprotein cholesterol
ABL: Abetalipoproteinemia
ER: Endoplasmic reticulum
PDI: Protein disulfide isomerase
NAFLD: Non-alcoholic fatty liver disease
CAD: Cardiovascular disease
AST: Aspartate transaminase
ALT: Alanine transaminase
HDL-C: High-density lipoprotein cholesterol
NEFA: Non-esterified fatty acid
BMI: Body mass index
MD: Molecular dynamics
