# Supplementary material for: MTTP-297H polymorphism reduced serum cholesterol but increased risk of non-alcoholic fatty liver disease-a cross-sectional study
Source: BMC Med Genet. 2015 Oct 12;16:93. doi: 10.1186/s12881-015-0242-6 (PMC4603340; doi:10.1186/s12881-015-0242-6)
Supplement: Additional file 1: — Risk impact and interaction of the MTTP genotypes on serum triglyceride. (DOCX 21 kb) [file 12881_2015_242_MOESM1_ESM.docx]

Additional file 1. Risk impact and interaction of the *MTTP* genotypes on serum triglyceride

| **Independent**  **variables** | **Parameter estimates (B)** | **95% Confidence interval**  **Lower vs Upper bound** | | ***P*** |
| --- | --- | --- | --- | --- |
| Age | -0.084 | -0.660 | 0.493 | 0.776 |
| Sex (male vs female) | 35.487 | -50.062 | 121.037 | 0.416 |
| BMI | 3.997 | 2.413 | 5.580 | **< 0.0001** |
| HOMA-IR | -15.173 | -19.138 | -11.207 | **< 0.0001** |
| Adipo-IR | 14.643 | 12.442 | 16.844 | **< 0.0001** |
| E98D (CC vs GG+GC) | -32.339 | -138.365 | 73.686 | 0.550 |
| I128T (CC vs TT+TC) | -21.727 | -60.817 | 17.362 | 0.276 |
| N166S (GG vs AA+AG) | 14.366 | -91.789 | 120.521 | 0.791 |
| Q297H (CC vs GG+GC) | 11.427 | -2.566 | 25.420 | 0.109 |

* Multiple linear regression analysis was applied using serum triglyceride as dependent variable adjusted by age, sex, BMI, HOMA-IR, Adipo-IR and *MTTP* genotypes based on significance in univariate analyses and regression models. (*P* < 0.05 indicates significant)
