# Supplementary material for: MTTP-297H polymorphism reduced serum cholesterol but increased risk of non-alcoholic fatty liver disease-a cross-sectional study
Source: BMC Med Genet. 2015 Oct 12;16:93. doi: 10.1186/s12881-015-0242-6 (PMC4603340; doi:10.1186/s12881-015-0242-6)
Supplement: Additional file 2: — Comparison of the biochemistry among the 10 haplotypes. (DOCX 22 kb) [file 12881_2015_242_MOESM2_ESM.docx]

Additional file 2. Comparison of the biochemistry among the 10 haplotypes

| **Independent variables** | **Parameter estimates (B)** | **95% Confidence**  **interval** | ***P*** |
| --- | --- | --- | --- |
| **Cholesterol** | | | |
| Age | 0.424 | 0.213 ~ 0.635 | <0.001 |
| 12121 | -7.177 | -13.345 ~- 1.008 | 0.0226 |
| 21232 | -6.757 | -13.266 ~ -0.248 | 0.0419 |
| 21233 | -11.035 | -19.347 ~ -2.723 | 0.0093 |
| **LDL-C** | | | |
| Sex (female vs male) | -11.143 | -18.848 ~ -3.437 | 0.0047 |
| 21233 | -13.789 | -23.708 ~ -3.870 | 0.0065 |
| **Non-HDL-C** | | | |
| Age | 0.648 | 0.401 ~ 0.896 | < 0.0001 |
| Sex (female vs male) | -13.941 | -21.418 ~ -6.464 | 0.003 |
| 21233 | -11.078 | -20.703 ~ -1.453 | 0.0241 |
| **HDL-C** | | | |
| Sex (female vs male) | 10.244 | 7.305 ~ 13.184 | <0.0001 |
| 22222 | 5.557 | 0.593 ~ 10.522 | 0.0283 |
| **BMI** | | | |
| Sex (female vs male) | -2.547 | -3.210 ~ -1.863 | <0.0001 |
| 13111 | 0.706 | -3.175 ~ -0.403 | 0.0115 |
| **AST** | | | |
| Sex (female vs male) | -4.271 | -5.983 ~ -2.560 | < 0.0001 |
| 11133 | 2.202 | 0.192 ~ 4.212 | 0.0318 |
| **ALT** | | | |
| Sex (female vs male) | -8.484 | -11.485 ~ -5.483 | <0.0001 |
| 11133 | 3.820 | 0.295 ~ 7.346 | 0.00337 |
| **NAFLD** |  |  |  |
| 11133 | 1.680 (OR) | 1.009~2.570 | 0.0168 |

The haplotype is composed and indicated sequentially as G-493T/E98D/I128T/N166S/Q297H. All of the haplotypes were simply indicated as number 12121(GG/GC/TT/AG/GG), 21232(GT/GG/TC/GG/GC), 21233(GT/GG/TC/GG/CC),22222(GT/GC/TC/AG/GC), 13111(GG/CC/TT/AA/GG), 11133(GG/GG/TT/GG/CC) referenced to control 11132 (GG/GG/TT/GG/GC).

Biochemistry of the reference haplotype 11132 (GG/GG/TT/GG/GC) was as following: total cholesterol 184.11±34.97 mg/dl; LDL-C 126.54±35.14, non-HDL-C 132.59±34.83 mg/dl, HDL-C 51.96±12.76 mg/dl, BMI 24.6±3.27 kg/m^2^, AST 17.88±6.66 IU/L, ALT 18.89±12.36 IU/L. NAFLD indicates non-alcoholic fatty liver disease. OR indicates odds ratio.
